# Machine Learning Backpropagation Prediction and Analysis of the Thermal Degradation of Poly (Vinyl Alcohol)

**DOI:** 10.3390/polym16030437

**Published:** 2024-02-05

**Authors:** Abdulrazak Jinadu Otaru, Zaid Abdulhamid Alhulaybi, Ibrahim Dubdub

**Affiliations:** Chemical Engineering Department, College of Engineering, King Faisal University, P.O. Box 380, Al Ahsa 31982, Saudi Arabia; zalhulaybi@kfu.edu.sa (Z.A.A.); idubdub@kfu.edu.sa (I.D.)

**Keywords:** poly (vinyl alcohol), TGA, experiment, machine learning

## Abstract

Thermogravimetric analysis (TGA) is crucial for describing polymer materials’ thermal behavior as a result of temperature changes. While available TGA data substantiated in the literature significantly focus attention on TGA performed at higher heating rates, this study focuses on the machine learning backpropagation analysis of the thermal degradation of poly (vinyl alcohol), or PVA, at low heating rates, typically 2, 5 and 10 K/min, at temperatures between 25 and 600 °C. Initial TGA analysis showed that a consistent increase in heating rate resulted in an increase in degradation temperature as the resulting thermograms shifted toward a temperature maxima. At degradation temperatures between 205 and 405 °C, significant depths in the characterization of weight losses were reached, which may be attributed to the decomposition and loss of material content. Artificial neural network backpropagation of machine learning algorithms were used for developing mathematical descriptions of the percentage weight loss (output) by these PVA materials as a function of the heating rate (input 1) and degradation temperature (input 2) used in TGA analysis. For all low heating rates, modelling predictions were observably correlated with experiments with a 99.2% correlation coefficient and were used to interpolate TGA data at 3.5 and 7.5 K/min, indicating trends strongly supported by experimental TGA data as well as literature research. Thus, this approach could provide a useful tool for predicting the thermograms of PVA materials at low heating rates and contribute to the development of more advanced PVA/polymer materials for home and industrial applications.

## 1. Introduction

Recent years have seen a significant rise in the use of polymeric and polymer-modified materials as the world’s population continues to grow. This has been achieved through continued harnessing and formulation of resources available within the context of man’s environment. As compared to ceramic or metallic structures, polymer materials have the advantage of being lighter, more resistant to water and grease and possessing multiplier characteristics of parts derived from the combination of monomers [1]. These combined and unique characteristics of polymer materials enabled their utilization in several applications, such as making polyethylene cups and plates, epoxy glue, polyurethane foam cushions, Teflon-coated cookware, fiber glass, and plastic bags [2]. Examples of polymeric materials include polyethylene, polypropylene, polystyrene, polyvinylchloride, Teflon, nylon, poly (methyl methacrylate), and poly (vinyl alcohol) [3].

Among the different examples of polymeric materials, poly (vinyl alcohol) is a focus of considerable interest due to wide applications in construction and woodworking, food packaging, cosmetics and biomedical industries [4,5]. In general, poly (vinyl alcohol) is abbreviated to PVA, and this material is considered a synthetic water-soluble linear polymer (hydrophilic characteristic) that forms copolymers of vinyl alcohol and vinyl acetate with idealized chemical formulas [CH2CH(OH)]n [6,7]. Abdullahi et al. [5] noted that in addition to PVA’s high tensile strength, other desirable properties of these materials allowed for a variety of applications in man’s daily life. These characteristics include good adhesion, good chemical resistance, film-forming ability, and biocompatibility. In many cases, the degradation of this material is attributed to weight changes, resulting from oxidation and decomposition reactions and from physical processes like desorption, vaporization, or sublimation [8,9,10]. Hence, it is critical to estimate its thermal degradability by measuring the weight changes in the material in response to temperature and heating rate (thermogravimetric analysis, TGA).

There is a considerable amount of research in the literature dealing with the thermal degradability of polymer materials, using either prediction or experimental methods. As an example, Varma and Sadhir [11] investigated the thermal properties of polyvinyl alcohol (PVA) and polyvinyl acetate (PVAc) with dynamic thermogravimetry. A direct correlation was found between the stability of PVAc and its molecular weight, whereas that of PVA decreased with decreasing molecular weight. According to Hayashi et al. [12], thermal degradation of PVA involves a complex and multiple process of chain scission, cross-linking, and dehydroxylation, all of which occur simultaneously and at varying temperatures and environmental conditions. Tsioptsias et al. [13] found that thermogravimetric analysis of physical cross-linked PVA films at carefully selected temperatures (50–600 °C) revealed an increasing number of hydrogen bonds, higher thermal stability, and a slower decomposition rate than raw PVA powder. In [14], the kinetics of thermal degradation of a mixed polymer composite comprising PVA, alcohol, starch, carboxymethyl cellulose, and clay (PVA/S/CMC/MMT) was studied experimentally. By applying the Ozawa method at heating rates between 7.5, 10, and 15, activation energies between 69 and 76 KJ/mol were obtained for decomposition temperatures ranging from 250 to 350 °C. In addition, PVA/S/CMC/MMT blends were reported to have high thermal stability when compared with PVA/S/CMC blends, indicating that the MMT material confers an advantage [14]. Thermogravimetric analysis of PVA nanofibrous membranes made via electro-spinning was examined by Ozturk et al. [15]. Their study [15] revealed that degradation of cross-linked PVA and pure PVA nanofibers starts at 240 °C and 290 °C, respectively, for 10 °C/min heating. The thermogravimetric analysis (TGA) and differential scanning calorimetry (DSC) of polyvinyl alcohol and starch (PVA–SF) fiber blends were investigated in [16]. The characteristic of cyclic hemiacetals provided by the starch was reported [16] to prevent thermal attacks on the polymer blends and, as such, their thermal behavior shifted to a higher temperature when degradation onset occurred as compared with values recorded for pure PVA materials. The study by Jadhav et al. [17] evaluated the thermal degradation of pure polyvinyl alcohol (PVA) and nanocomposite (polyvinyl alcohol [PVA]–polypyrrole [Ppy]–gold [Au]) films that were synthesized by in situ chemical oxidants with variable gold particle loadings. PVA–Ppy and PVA–Ppy–AU nanocomposites showed better thermal stability without rapid degradation after an experiment conducted at 10 °C/min heating and degradation temperatures between 25 and 900 °C. According to [17], the gold particles served as a mass transport barrier that inhibited PVA–Ppy degradation.

Researchers have examined the thermal stability of polymer materials through kinetic studies, resulting in kinetic triplet estimates: reaction model, pre-exponential factor (A) and activation energy [18,19,20]. Studying polymer materials from a kinetic perspective offers numerous advantages, including understanding the rate at which the reactant decreases or the product is formed, the mechanism of reactions at different conversion rates, the number of reaction steps, the number of reactions, the duration of reactions, cross-linking polymerization with diffusion control, and competing or independent steps. However, it is dependent on known thermograms to determine these kinetic triplets, which help reveal a depth of understanding of polymer thermal stability. In other words, experimentation is essential to the estimation of these kinetic parameters. Recently, machine learning artificial neural networks (ML-ANN) have also been adopted by analogous research in polymer and material engineering. Both academics and researchers in tertiary institutions and industry have applied ML-ANN to describe patterns in experimental data of polymeric materials, typically their TGA analysis. For example, Dubdub and Al-Yaari [21] applied a feed-forward backpropagation ANN approach with ten (10) neurons, two hidden layers, and logsig–logsig transfer function to forecast TGA data of low-density polyethylene for heating rates of 5, 10, 20 and 40 K/min. Their modelling data correlated well with experiments, and a correlation value of $R^{2}\to 0.9999$. Kianfar et al. [22] reported a correlation value of $R^{2}\to 0.9913$ between experimentally measured and modelled TGA data of alginate/polyvinyl alcohol using the Levenberg–Marquardt algorithm of artificial neural networks (ANN). Using a multilayer perceptron neural network (MLP), Araujo et al. [23] assessed the contribution of several kinetic models to accurately describe TGA experimental data for chitosan biopolymers at different heating rates: 2.5, 5.0, 7.5 and 10 °C/min. Their predictions nearly overlapped experiments and categorized contributions from volume contraction and surface area contraction models.

Many studies have been conducted on TGA analysis of pure poly (vinyl alcohol) or blends of it with other polymers, but few predictions have been made for pure PVA and at low heating rates. Accordingly, this study provides a machine-learning backpropagation description of the thermal degradation of PVA materials based on TGA measurements conducted in a laboratory. The first aspect of this study involves examining and providing TGA data and procedures for experiments involving thermal degradation at low heating rates, typically 2, 5 and 10 K/min. The second aspect of this study involves mathematical formulations and discussions of predictions obtained from machine learning backpropagation.

## 2. TGA Experimental Procedure and Data

The thermogravimetric analysis (TGA) experiment was conducted according to the procedures described in [1]. In brief, the thermal decomposition of the PVA materials was studied in a thermogravimetric analyzer consisting of three different components supplied from Mettler Toledo. For this study, the poly(vinyl alcohol) used was that of Industrial and Scientific grade 1788L powder with the following characteristics: molecular weight of 74,800 kDa, 88% alcoholysis and 1700 polymerization degree. Samples in powder form of this material were used for this experiment, with each sample placed on the TGA pan and weighted at the beginning. A 40 mL/min nitrogen (N_2_) gas was injected into the device’s oven to study decomposition in an inert environment at three different heating rates of 2, 5, and 10 K/min. Purging nitrogen gas into the TGA chamber makes the surrounding area inert and prevents weight loss due to oxidation [11]. All three test samples were labelled with PVAx labels throughout this paper to indicate their different heating rates. Decomposition weight changes in materials were studied over time at decomposition temperatures ranging between 25 and 600 °C. TGA data for weight loss of these PVA samples as well as temperature data for degradation were extracted from the TGA device and prepared for modelling.

**Figure 1:**
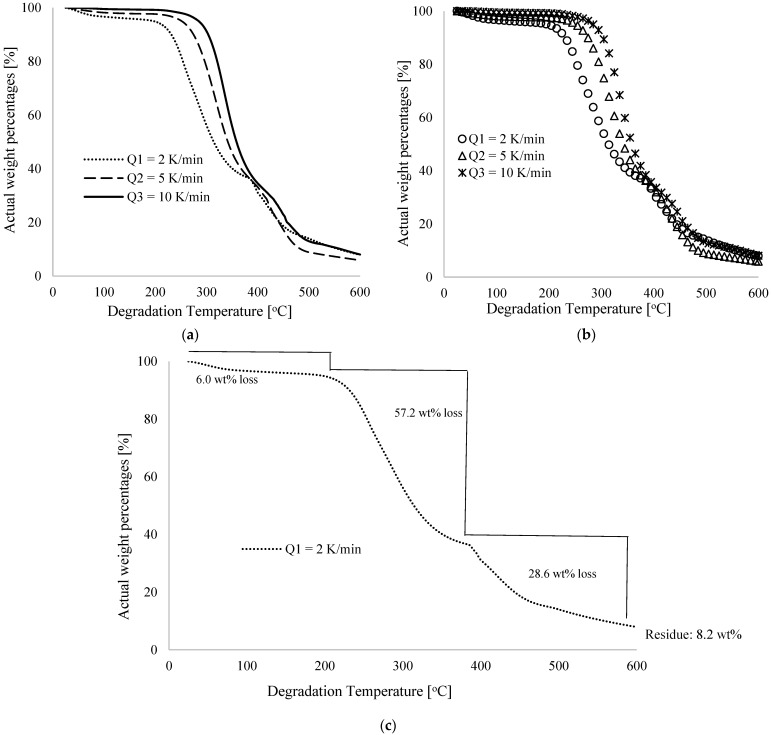
Characteristic thermograms showing typical plots of actual weight percentages [wt%] against degradation temperature [°C] for (**a**) raw experimental data for different low heating rates of 2.0, 5.0 and 10.0 K/min, (**b**) selected experimental data for temperatures of 25 to 600 °C at 10 °C difference and (**c**) percentage weight losses at a 2 K/min heating rate.

Figure 1a shows thermograms of percentage weight losses by these samples against degradation temperature performed at different low heating rates of 2, 5, and 10 K/min. In Figure 1b, some selected datasets along the various thermograms (from 25 to 600 °C with an equal scale difference of 10 °C) are extracted for use in the backpropagation modelling approach (to be discussed later), whereas Figure 1c shows the percentage weight loss for a heating rate of 2 K/min along the degradation temperature. Figure 1 shows that the percentage weight loss changes non-linearly with degradation temperature and this supports the generalized behavior of materials in TGA analysis described in [24,25,26]. The weight losses observed in the pure PVA materials used in this study can be attributed to dehydration (evaporation of water), nitrogen gas loss, and decomposition of the polymer materials as the degradation temperature increases [27]. The research described in [9,15] indicated that variations in furnace heating rate, furnace temperature, and sample characteristics (weight and particle size) contributed to this non-linear decrease in polymeric material weight percentage as degradation temperature increased. This study utilized nitrogen gas to keep the oven inert to reduce the decomposition temperature [27,28] and minimize the exposure of the polymer sample to atmospheric air. As opposed to an oxidative environment largely dominated by air, the decomposition temperature is often reduced in a TGA system with an inert environment containing nitrogen (N_2_), carbon dioxide (CO_2_), and argon (Ar) [27].

Figure 1a illustrates the increase in degradation temperature as the furnace heating rate is increased. At 2 K/min, the onset of degradation observably started immediately after room temperature of 25 °C for sample 1, which weighs 7.80 mg (100 wt%). As shown in Figure 1c, the percentage weight loss for this sample at 30, 100, 210, 380, and 590 °C was 0.16, 3.4, 6.0, 63.2 and 91.4, respectively, bringing the final weight of the first sample, when heated at 2 K/min, to 0.62 mg (8.2 wt%) at the maximum degradation temperature recorded (600 °C). TGA curves shifted to higher temperatures when heating rates were 5 and 10 K/min with similar non-linear trends. Detectable degradation began at 248 °C (1.9 wt% loss) and 262 °C (2.0 wt% loss) for heating rates of 5 and 10 K/min, respectively. At 380 °C, percentage weight losses were, respectively, 62.8 wt% and 60.0 wt%, indicating a gradual decline with increasing heating rate for these materials. However, the highest heating rate of 10 K/min increased the degradation time the most, followed by 5 and 2 K/min, respectively. Further, the initial weight of the sample recorded at 5 K/min was 7.69 mg (100 wt%), which was ultimately reduced to 0.45 mg (5.7 wt%) at 600 °C as the final weight of the residue after the TGA experiment. At a final degradation temperature of 600 °C, the weight of the sample was reduced from 7.10 mg (100 wt%) to 0.57 mg (8.0 wt%) at a rate of 10 K/min. Across all thermograms for the three different heating rates, significant weight losses are observed at mid-range degradation temperatures, typically between 205 and 405 °C, which could be explained by the sudden loss of weight caused by the decomposition of carbon–hydrogen molecules in the PVA materials. A key insight is that at a low degradation temperature, typically between 25 and 100 °C, the percentage weight loss in these materials can be attributed to the dehydration of the pure PVA material, whereas after this temperature, the decomposition of the material is responsible for the loss [14,15].

## 3. Machine Learning Backpropagation Neural Network and Data

**Figure 2:**
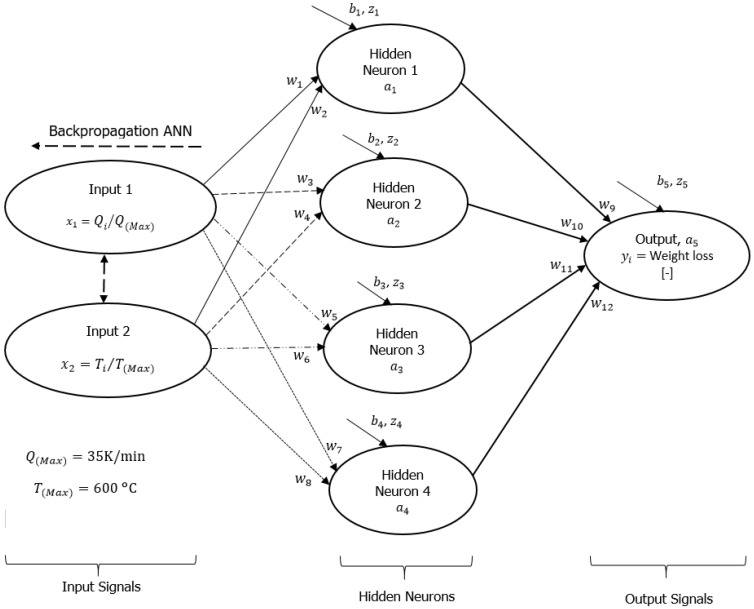
Machine learning backpropagation network analysis framework showing typical input, hidden and output neurons.

The machine learning backpropagation technique of the artificial neural network (ML-ANN) adopted for this study was first performed by formulating mathematical models describing the input and output variables for the TGA experiments for pure PVA materials, followed by trainings of the experimental datasets using the formulated ANN models. Figure 2 illustrates the ML-ANN framework that guided the formulation of the mathematical models. This figure shows that two input neurons and an output signal are linked by four hidden neurons ($a_{1}-a_{2}$), twelve synaptic weights (nerves, [$w_{i}$]) and five biases ($b_{i}$). Weight loss of the PVA materials during the TGA experiment represents the output signal ($y_{i}$), while heating rates ($Q_{i}$) and degradation temperatures ($T_{i}$) represent inputs 1 and 2, respectively. A preliminary assessment and sorting of the raw data from the TGA experiment was performed by selecting some data points along the thermograms for the three different heating rates and considering the initial and final degradation temperatures (25 and 600 °C) used in the experiment. As discussed in [29,30,31], the introduction of the hidden neurons (layers) into the framework in Figure 2 was performed to improve convolution and non-linearity between input and output signals that have been determined experimentally.

To accurately represent datasets for machine learning training, input and output variables are usually converted to values between 0 and 1. In this context, Sigmoid [32] is selected as the activation function in Equation (1b), which is a test function of the sum weight (Equation (1a)) and overall cost function of the ML-ANN framework. According to Panneerselvam [32], the activation functions in machine learning are chosen to compute synaptic weights and biases that most accurately describe reality (the real signal), which should be monotonic, differentiable, and easily converging. In general, they can be divided into linear and non-linear activation functions [32]. While there are a variety of non-linear activation functions, the Sigmoid function was selected because of its simplicity in allowing complex data combinations and its ability to output data between 0 and 1 when input data is given. By dividing by the maximum values, the real inputs (heating rates and degradation temperatures) and outputs (weight loss) can be converted to values between 0 and 1. Because of this, the percentage weight losses of the PVA materials were converted into their fractional values ($y_{i}$). The input heating rates were also divided by a maximum value of 35 K/min ($Q_{max}$) and served as input 1 ($x_{1}$), whereas temperature data were divided by the maximum degradation temperature of 600 °C used in this experiment and served as input 2 ($x_{2}$). Though the TGA experiment was conducted at low heating rates between 2 and 10 K/min, 35 K/min was selected as the maximum heating rate for the ML-ANN modelling so that the mathematical formulations accurately capture the behavior of pure PVA materials under higher heating rates (extrapolation). A detailed mathematical formulation for this framework (Figure 2), along with the cost optimization function, is available in the Supplementary Material.

(1a) $$z_{5}=b_{5}+w_{9}\cdot a_{1}+w_{10}\cdot a_{2}+w_{11}\cdot a_{3}+w_{12}\cdot a_{4}$$

(1b) $$a_{5}=\sigma^{\prime }\left(z_{5}\right)=\frac{1}{\left(1+e^{-z_{5}}\right)}$$

(1c) $$C={\left(y_{i}-a_{5}\right)}^{2}$$

where $z_{5}$ is the sum weight, $w_{i}$ is the synaptic weight, $\sigma^{\prime }\left(z_{5}\right)$ is the logistic function of the sum weight, $a_{i}$ is the activation function or predicted output signal, $y_{i}$ is the real/experimental output and C is the cost function.

**Figure 3:**
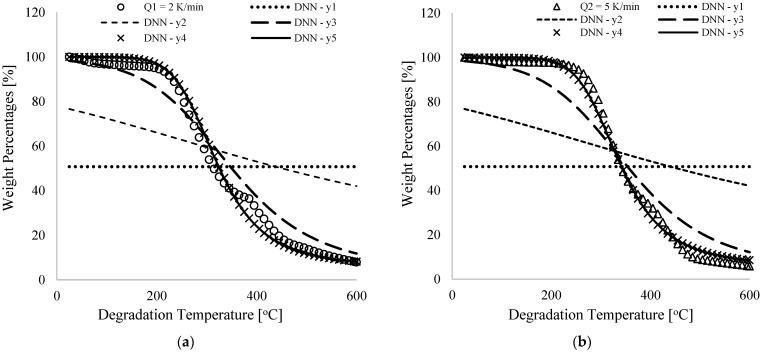

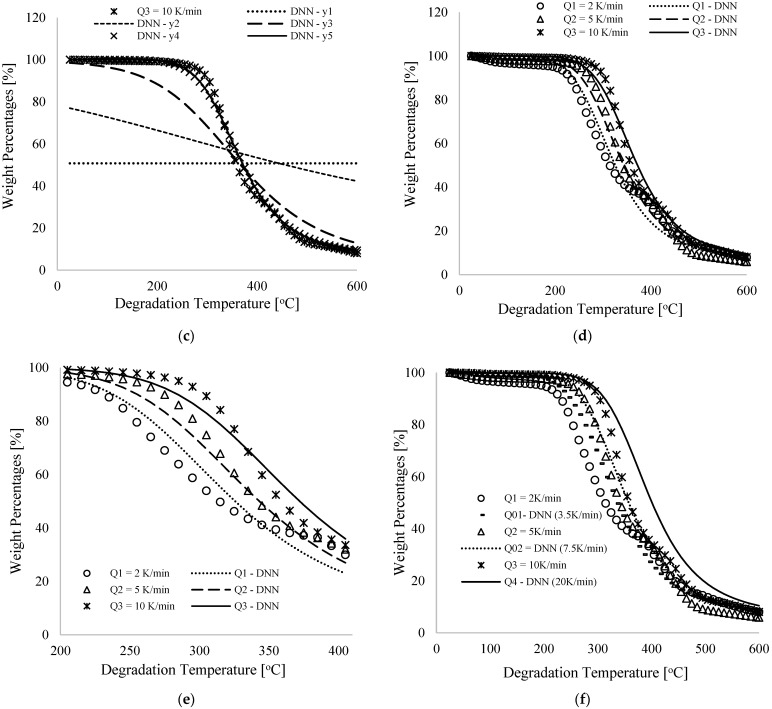
Experimental and DNN modelling data of PVA weight percentages against degradation temperature [°C] for heating rates of (**a**) 2 K/min; (**b**) 5 K/min; (**c**) 10 K/min; (**d**) 2, 5, and 10 K/min; (**e**) 2, 5, and 10 K/min at a temperature of 205–405 °C; and (**f**) including interpolated and extrapolated data for heating rates between 3.5, 7.5 and 20 K/min.

Experimental and DNN predictions are shown in Figure 3 for percentage weight loss (%) for pure PVA materials against degradation temperature (°C) at 2, 5 and 10 K/min. Table 1 presents numerically simulated arbitrary constants ($b_{i}\;and\;w_{i}$), linearity rates ($k_{P}$), reduced cost ($\sum_{i=1}^{N}C$) and percentage correlations ($R^{2}$) derived from comparing experiments with predictions. As shown in Figure 3a, different values of DNN predicted data have been obtained for the thermogram obtained at 2K/min heating rate at the beginning of the training (DNN–y_1_), mid-level trainings (DNN–y_1_, DNN–y_3_ and DNN–y_4_), and finally training (DNN–y_5_). Figure 3b,c illustrate thermogram plots for heating rates of 5 and 10 K/min. In Table 1, equal values of variables (0.01) were chosen at the beginning of the training and this resulted in unchanging values (typically, in the middle of the experiment) with increasing degradation temperatures as shown in Figure 3a–c. For a constant linearity rate of 5.0, continuous training of the DNN framework in Figure 2 resulted in changes associated with the originally selected synaptic weights and biases from the input to the output neurons as predicted by the DNN, illustrated in Table 1 by increasing correlation coefficients ($R^{2}$). According to this table, 99.2% correlation was achieved for the final trained DNN data. DNN predictions and experiments overlap completely (Figure 3) for all the heating rates considered herein as cost functions decrease from an initial value of 26.4 to 0.183 as shown in Table 1.

Across input and hidden neurons, the tabulated data in Table 1 indicate that synaptic weights associated with heating rates ($w_{1},\;w_{3}\;and\;w_{5}$) and degradation temperature ($w_{2},\;w_{4}\;and\;w_{6}$) were positive and negative, and the hidden neurons’ biases (i.e., $b_{1}=b_{2}=b_{3}=b_{5}=0.421$) were equal. The biases pointed to an offset of the training data as well as an increase in flexibility [32], but the synaptic weights showed an observable increase along with their signs as training progressed. Input 1 to hidden neurons are linked by positive synaptic weights, indicating that increasing the heating rate will cause an increase in the percentage weight loss (output signal). As seen in Figure 1, for a 105 °C degradation temperature, 96.6, 98.1 and 99.5% weight losses are obtained for heating rates of 2, 5 and 10 K/min, respectively. The degradation temperature at 405 °C was calculated at 29.9, 32.0, and 33.6%, respectively, for the different heating rates. Similarly, the DNN predicted data for heating rates of 2, 5 and 10 K/min and extrapolated DNN data for higher heating rates (to be discussed later) showed similar trends. Numerically simulated negative values for synaptic weights linking input 2 and hidden neurons indicate that increasing degradation temperature decreases percentage weight loss by pure PVA materials. These trends can be seen in all the thermograms obtained for the different heating rates considered in both TGA analysis and DNN prediction presented in Figure 1. Table 1 also shows that the synaptic weights linking the hidden neurons with the output signal were equal. This can be attributed to the equal values in synaptic weights and biases received by the hidden neurons ($a_{1}-a_{4}$) from the input neurons ($x_{1}\;and\;x_{2}$) and shows that all the hidden neurons were equally important in fully describing the convolution and non-linearity in the experimental datasets for the different heating rates of the pure PVA materials.

Figure 3c shows a nearly complete overlap between DNN predictions and experiments for mid-range degradation temperatures, typically between 205 and 405 °C, which has the highest weight loss percentage for the varied heating rates considered here. Furthermore, the final trained synaptic weights and biases were used to predict the weight loss for pure PVA material at interpolated and projected heating rates of 3.5 and 7.5 and 20K/min for the selected degradation temperature range between 25 and 600 °C, as shown by Figure 3f. Different heating rates resulted in comparable thermograms, which also showed a consistent shift towards temperature maxima with increasing heating rates. The interpolated thermogram at 3.5 K/min, for example, shifted between experimental and interpolated TGA data at 2 and 5 K/min heating rates. In addition, it was observed that predicted thermograms at 7.5 K/min heating rates switched between experimental thermograms at 5 and 10 K/min, proving the validity of this model for predicting PVA thermograms at low heating rates. The extrapolated thermogram in Figure 3f indicates the onset of degradation at 275 K/min, slightly higher than that at 10 K/min. The numerically predicted extrapolated thermograms at 20 K/min show an increase over current experiments at 2–10 K/min heating rates, and this supports an analogous trend [9,10,11,12,13,14,15] on the TGA analysis of polymer materials, which shows that degradation starts at a higher temperature when the heating rate is increased. Extending the validity or limitation of these models in predicting the thermal behavior of PVA material beyond 10 K/min, however, will require more experimental analysis to confirm their validity. Hence, a future study is proposed to incorporate additional inputs such as polymer degradation time, composition and expanded heating rates for different classes of selected polymer and co–polymer materials into machine learning models. A global model could be developed using this approach, and this research is already in consideration by this group.

A careful approach was used to formulate the mathematical models (Supplementary Data), utilizing the general artificial neural network model in Equation 1 and working through the carefully drawn framework of Figure 2 from output to input signals (backpropagation). As shown in Figure 2, this framework consists of two input neurons, four hidden neurons (single layer), and one output neuron linked by twelve synaptic weights and five biases. In this case, the number of neurons or layers determines the hyperparameters of the machine learning model, which were selected based on prior experience with deep neural analysis and an analysis of the relationships between input and output experimental datasets. According to the leading author of this group [30], increasing this hyperparameter (that is, adding neurons or layers) results in increased accuracy (reduced true error) and convergence, but at the cost of an increased mathematical formulation time and model complexity. By reducing the hyperparameter, the true error between the output signal and reality increases, as well as the convergent time. However, the initial framework in Figure 2, followed by mathematical models, produced final output signals which completely overlap reality and also reduced the overall cost function of the models to nearly zero. A linearity rate of 5.0 was used in training these mathematical models in Microsoft Excel with code written in its Visual Basic for Applications. The datasets were continuously trained at a computational and copying time step of 1 s, followed by fast tracking to a zero time step for about seven consecutive days in order to achieve the desired and nearly zero cost function. The thermograms for three heating rates were all used at once during the training of these models, which resulted in millions of iterations (epochs) over the seven days. This means that the learning algorithm for the formulated models required millions of epochs in order to improve the agreement between simulations and experiments and result in trained synaptic weights and biases. According to Table 1 and Figure 3, predictions of synaptic weights and biases for the initial guess values were poor, with an overall cost function of 26.427 indicating a wide margin between predictions and reality. After a series of iterations, the learning algorithm (mathematical models) in the Supplementary Data begins to understand these data through the network in Figure 2. As the overall cost function approaches zero (0.183, see Table 1), predictions nearly overlap experiments (99.2%). Figure 2 shows that a single hidden layer consisting of four neurons was able to achieve this close correlation between predictions and experiments. However, a more complex framework involving more than a single layer and several neurons could still provide us with similar or greater correlations, but at a cost of computational time in the development of learning algorithms, resulting in the attainment of more synaptic weights and biases. In contrast, fewer hidden neurons could have been used in Figure 2, but at the cost of computational time and accuracy. This is the called Kolmogorov complexity [33], which identifies the length of the shortest computer program (learning algorithm in this case) that produces a specified output. 

## 4. Conclusions

In this study, experimental and machine learning backpropagation formulations and analyses of TGA data obtained for degradation of PVA materials are presented for differing heating rates of 2, 5 and 10 K/min and degradation temperatures ranging from 25 to 600 °C. PVA degradation temperatures increased with increasing heating rates, resulting in higher percentage weight losses at degradation temperatures between 205 and 405 °C. Training the experimental datasets with the formulated backpropagation neural network models yielded a 99.2% correlation coefficient between predictions and experiments. With increasing heating rates, the numerically simulated DNN-trained synaptic weights and biases showed an increase in the percentage weight loss of pure PVA polymer materials, whereas, with increasing degradation temperatures, the percentage weight loss decreased. The trained arbitrary constants were also used to extrapolate thermograms for higher heating rates, typically between 15 and 35 K/min, and similar trends were observed when compared to TGA experimental data published in the literature. The extrapolated data may still require experimental validation and may form the basis for further research. Nevertheless, the trained arbitrary constants and learning algorithm could provide a framework for understanding and predicting polymer materials’ thermograms, allowing them to be designed and applied ideally in both domestic and industrial settings.

## Figures and Tables

**Table 1:** Tabular representation of DNN computed values of arbitrary constant ($w_{i}\;\&{\;b}_{i}$), linearity rate ($k_{P}$), reduced cost function ($\sum_{i=1}^{N}C$) and percentage correlations ($R^{2}$).

	**b_1_**		**w_1_**		**w_2_**		**b_2_**		**w_3_**		**w_4_**		**b_3_**		**w_5_**		**w_6_**	
**y_1_**	0.010		0.010		0.010		0.010		0.010		0.010		0.010		0.010		0.010	
**y_2_**	0.068		0.066		−1.454		0.068		0.066		−1.454		0.068		0.066		−1.454	
**y_3_**	0.790		0.350		−2.838		0.790		0.350		−2.838		0.790		0.350		−2.838	
**y_4_**	0.904		1.695		−5.022		0.904		1.695		−5.022		0.904		1.695		−5.022	
**y_5_**	0.421		1.591		−4.699		0.421		1.591		−4.699		0.421		1.591		−4.699	

	**b_4_**	**w_7_**		**w_8_**		**b_5_**		**w_9_**	**w_10_**	**w_11_**		**w_12_**		**k_p_**		$\sum_{i=1}^{N}C$		**R^2^ [%]**
**y_1_**	0.010	0.010		0.010		0.010		0.010	0.010	0.010		0.010		5.000		26.427		33.174
**y_2_**	0.068	0.066		−1.454		−1.324		1.249	1.249	1.249		1.249		5.000		13.763		47.732
**y_3_**	0.790	0.350		−2.838		−3.282		2.734	2.734	2.734		2.734		5.000		1.376		82.616
**y_4_**	0.904	1.695		−5.022		−2.740		4.538	4.538	4.538		4.538		5.000		0.187		98.453
**y_5_**	0.421	1.591		−4.699		−2.799		6.073	6.073	6.073		6.073		5.000		0.183		99.157

## Data Availability

Data will be provided upon request.

## Supplementary Materials

The following supporting information can be downloaded at: https://www.mdpi.com/article/10.3390/polym16030437/s1.
