# Supplementary material for: Machine Learning Backpropagation Prediction and Analysis of the Thermal Degradation of Poly (Vinyl Alcohol)
Source: Polymers (Basel). 2024 Feb 5;16(3):437. doi: 10.3390/polym16030437 (PMC10856943; doi:10.3390/polym16030437)
Supplement: Supplementary file 1 [file polymers-16-00437-s001.zip › polymers-2818539-supplementary.pdf]

Mathematical Formulation of the PVA Thermograms for 2, 5, and 10 K/min Heating Rates

To formulate the mathematical models for the framework in Figure 2, it is necessary to start with the general mathematical expressions of artificial neural networks (ANNs) [28]. These expressions relate the predicted (a_5) and real (y_i) output signals with the inputs and hidden neurons corresponding to them. The following are mathematical expressions formulating the output through hidden neurons to input neurons (backpropagation).

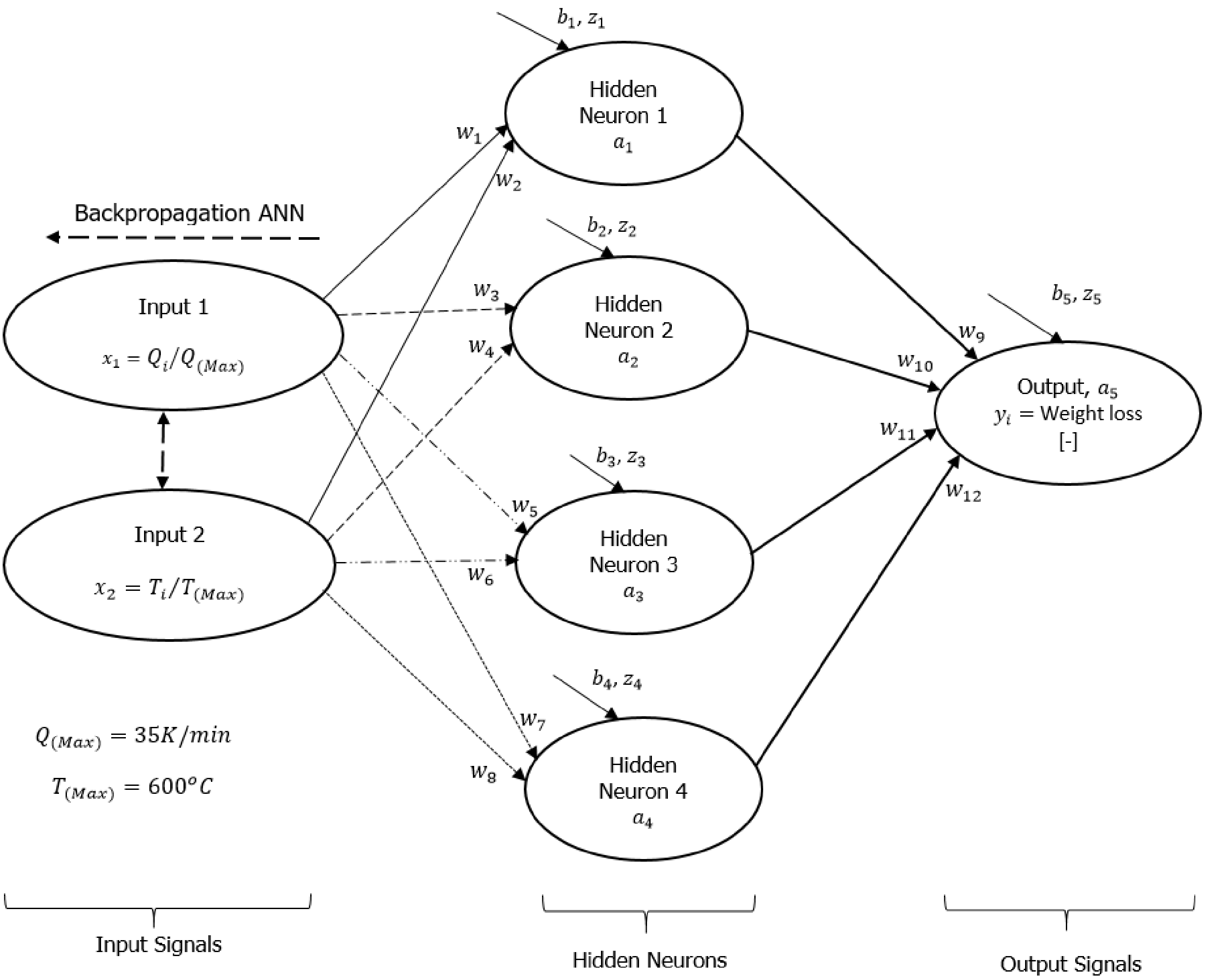

Figure S1. Machine learning backpropagation network analysis framework showing typical input, hidden and output neurons.

The sum of the weights of the output 5 (z_5) using Figure 2 is expressed as a function of bias (b_5), weights ($w_9 - w_{12}$) and activation of the hidden neurons ($a_1 - a_4$) as shown by Equation 1a.

$$z_5 = b_5 + w_9.a_1 + w_{10}.a_2 + w_{11}.a_3 + w_{12}.a_4 \quad \text{Eqn 1a}$$

The logistic function of output 5 [$\sigma'(z_5)$] also known as the Sigmoid activation (a_5) or predicted signal is expressed as

$$a_5 = \sigma'(z_5) = \frac{1}{(1+e^{-z_5})} \quad \text{Eqn 1b}$$

And the cost function (C) is expressed as

$$C = (y_i - a_5)^2 \qquad \text{Eqn 1c}$$

The logistic functions and sum weight for the four hidden neurons are expressed as follows:

$$a_1 = \sigma'(z_1) = \frac{1}{(1+e^{-z_1})} \ \& \ z_1 = b_1 + w_1.x_1 + w_2.x_2 \qquad \text{Eqn 1d}$$

$$a_2 = \sigma'(z_2) = \frac{1}{(1+e^{-z_2})} \ \& \ z_2 = b_2 + w_3.x_1 + w_4.x_2 \qquad \text{Eqn 1e}$$

$$a_3 = \sigma'(z_3) = \frac{1}{(1+e^{-z_3})} \ \& \ z_3 = b_3 + w_5.x_1 + w_6.x_2 \qquad \text{Eqn 1f}$$

$$a_4 = \sigma'(z_4) = \frac{1}{(1+e^{-z_4})} \ \& \ z_4 = b_4 + w_7.x_1 + w_8.x_2 \qquad \text{Eqn 1g}$$

where $b_1 - b_5$ and $w_1 - w_{12}$ are arbitrarily constants needed for predicting the output signal. Obtaining these values could be performed by trial and error, but this could be time consuming and tedious. Cost optimization is a more preferable approach and is described and mathematically expressed below:

1. How much does cost depend on output 5?

Since backpropagation ANN requires starting from the output data [26-28], the connections between the hidden and output layers can be referred to as linear, which is why single-layer neural networks (SNN) will be used to perform machine learning algorithms. As a result, a change in bias (Δb) and weight (Δw) is needed for the framework to be trained as shown in Figure 2 with Equation 2a.

$$\Delta b = -k_P \frac{\partial C}{\partial b} \quad \text{and} \quad \Delta w = -k_P \frac{\partial C}{\partial w} \qquad \text{Eqn 2a}$$

where k_P is the learning rate, which is a value chosen for the training of the network in order to estimate the arbitrary constants.

$$\left(\frac{\partial C}{\partial a_5}\right) = 2(a_5 - y); \quad \left(\frac{\partial a_5}{\partial z_5}\right) = \sigma'(z_5) = \frac{e^{z_5}}{(1+e^{z_5})^2}; \quad \left(\frac{\partial z_5}{\partial w_9}\right) = a_1; \left(\frac{\partial z_5}{\partial w_{10}}\right) = a_2; \quad \left(\frac{\partial z_5}{\partial w_{11}}\right) = a_3; \left(\frac{\partial z_5}{\partial w_{12}}\right) = a_4 \ \text{and} \ \left(\frac{\partial z_5}{\partial b_5}\right) = 1. \qquad \text{Eqn 2b}$$

$$\left(\frac{\partial C}{\partial b_5}\right) = \left(\frac{\partial C}{\partial a_5}\right)\left(\frac{\partial a_5}{\partial z_5}\right)\left(\frac{\partial z_5}{\partial b_5}\right) \qquad \text{Eqn 2c}$$

$$\left(\frac{\partial C}{\partial w_9}\right) = \left(\frac{\partial C}{\partial a_5}\right)\left(\frac{\partial a_5}{\partial z_5}\right)\left(\frac{\partial z_5}{\partial w_9}\right) \qquad \text{Eqn 2d}$$

$$\left(\frac{\partial C}{\partial w_{10}}\right) = \left(\frac{\partial C}{\partial a_5}\right)\left(\frac{\partial a_5}{\partial z_5}\right)\left(\frac{\partial z_5}{\partial w_{10}}\right) \qquad \text{Eqn 2e}$$

$$\left(\frac{\partial C}{\partial w_{11}}\right) = \left(\frac{\partial C}{\partial a_5}\right)\left(\frac{\partial a_5}{\partial z_5}\right)\left(\frac{\partial z_5}{\partial w_{11}}\right) \qquad \text{Eqn 2f}$$

$$\left(\frac{\partial C}{\partial w_{12}}\right) = \left(\frac{\partial C}{\partial a_5}\right)\left(\frac{\partial a_5}{\partial z_5}\right)\left(\frac{\partial z_9}{\partial w_{12}}\right) \quad \text{Eqn 2g}$$

Hence: $\Delta b_5 = -k_P\left(\sum_{i=1}^{N}\left(\frac{\partial C}{\partial b_5}\right)\right)/N$ & $b_{5(New)} = b_{5(old)} + \Delta b_5$ Eqn 2h

$\Delta w_9 = -k_P\left(\sum_{i=1}^{N}\left(\frac{\partial C}{\partial w_9}\right)\right)/N$ & $w_{9(New)} = w_{9(Old)} + \Delta w_9$ Eqn 2i

$\Delta w_{10} = -k_P\left(\sum_{i=1}^{N}\left(\frac{\partial C}{\partial w_{10}}\right)\right)/N$ & $w_{10(New)} = w_{10(Old)} + \Delta w_{10}$ Eqn 2j

$\Delta w_{11} = -k_P\left(\sum_{i=1}^{N}\left(\frac{\partial C}{\partial w_{11}}\right)\right)/N$ & $w_{11(New)} = w_{11(Old)} + \Delta w_{11}$ Eqn 2k

$\Delta w_{12} = -k_P\left(\sum_{i=1}^{N}\left(\frac{\partial C}{\partial w_{12}}\right)\right)/N$ & $w_{12(New)} = w_{12(Old)} + \Delta w_{12}$ Eqn 2l

2. Cost of output 5 on the hidden neurons

In this stage, the cost is estimated by estimating how much the hidden activations affect the cost, $a_1 - a_4$. The mathematical expression for estimating this type of cost function is given by Equation 3a.

$\left(\frac{\partial C}{\partial a_h}\right) = \left(\frac{\partial C}{\partial a_j}\right)\left(\frac{\partial a_j}{\partial z}\right)\left(\frac{\partial z}{\partial a_h}\right)$ where $\left(\frac{\partial z}{\partial a_h}\right) = w$ Eqn 3a

When Equation 3a is applied to the hidden neurons in Figure 2, the following equations are obtained:

$\left(\frac{\partial C}{\partial a_1}\right) = \left(\frac{\partial C}{\partial a_5}\right)\left(\frac{\partial a_5}{\partial z_5}\right)\left(\frac{\partial z_5}{\partial a_1}\right)$ where $\left(\frac{\partial a_5}{\partial z_5}\right) = \sigma'(z_5) = \frac{e^{z_5}}{(1+e^{z_5})^2}$ & $\left(\frac{\partial z_5}{\partial a_1}\right) = w_9$ Eqn 3b

$\left(\frac{\partial C}{\partial a_2}\right) = \left(\frac{\partial C}{\partial a_5}\right)\left(\frac{\partial a_5}{\partial z_5}\right)\left(\frac{\partial z_5}{\partial a_2}\right)$ where $\left(\frac{\partial z_5}{\partial a_2}\right) = w_{10}$ Eqn 3c

$\left(\frac{\partial C}{\partial a_3}\right) = \left(\frac{\partial C}{\partial a_5}\right)\left(\frac{\partial a_5}{\partial z_5}\right)\left(\frac{\partial z_5}{\partial a_3}\right)$ where $\left(\frac{\partial z_5}{\partial a_3}\right) = w_{11}$ Eqn 3d

$\left(\frac{\partial C}{\partial a_4}\right) = \left(\frac{\partial C}{\partial a_5}\right)\left(\frac{\partial a_5}{\partial z_5}\right)\left(\frac{\partial z_5}{\partial a_4}\right)$ where $\left(\frac{\partial z_5}{\partial a_4}\right) = w_{12}$ Eqn 3e

3. The cost function on inputs 1 and 2

By adopting the single-layer neural network (SNN), the hidden neurons can be trained to depend on inputs 1 and 2. In other words, input neurons and hidden layers are assumed to have a linear relationship. Hence, the mathematical relationship for these cost functions can be obtained from Equation 4 below at this stage of the mathematical formulation.

$$\frac{\partial C}{\partial w} = \left(\frac{\partial C}{\partial a}\right)\left(\frac{\partial a}{\partial z}\right)\left(\frac{\partial z}{\partial w}\right); \quad \frac{\partial C}{\partial b} = \left(\frac{\partial C}{\partial a}\right)\left(\frac{\partial a}{\partial z}\right)\left(\frac{\partial z}{\partial b}\right)$$ Eqn 4

Inputs connected to hidden neuron 1

$$\left(\frac{\partial C}{\partial b_1}\right) = \left(\frac{\partial C}{\partial a_1}\right)\left(\frac{\partial a_1}{\partial z_1}\right)\left(\frac{\partial z_1}{\partial b_1}\right) \text{ where } \left(\frac{\partial z_1}{\partial b_1}\right) = 1 \;\&\; \left(\frac{\partial a_1}{\partial z_1}\right) = \sigma'(z_1) = \frac{e^{z_1}}{(1+e^{z_1})^2}$$ Eqn 5a

$$\left(\frac{\partial C}{\partial w_1}\right) = \left(\frac{\partial C}{\partial a_1}\right)\left(\frac{\partial a_1}{\partial z_1}\right)\left(\frac{\partial z_1}{\partial w_1}\right) \text{ where } \left(\frac{\partial z_1}{\partial w_1}\right) = x_1$$ Eqn 5b

$$\left(\frac{\partial C}{\partial w_2}\right) = \left(\frac{\partial C}{\partial a_1}\right)\left(\frac{\partial a_1}{\partial z_1}\right)\left(\frac{\partial z_1}{\partial w_2}\right) \text{ where } \left(\frac{\partial z_1}{\partial w_2}\right) = x_2$$ Eqn 5c

Hence: $\Delta b_1 = -k_P\left(\sum_{i=1}^{N}\left(\frac{\partial C}{\partial b_1}\right)\right)/N \quad \& \quad b_{1(New)} = b_{1(Old)} + \Delta b_1$ Eqn 5d

$\Delta w_1 = -k_P\left(\sum_{i=1}^{N}\left(\frac{\partial C}{\partial w_1}\right)\right)/N \quad \& \; w_{1(New)} = w_{1(Old)} + \Delta w_1$ Eqn 5e

$\Delta w_2 = -k_P\left(\sum_{i=1}^{N}\left(\frac{\partial C}{\partial w_2}\right)\right)/N \quad \& \; w_{2(New)} = w_{2(Old)} + \Delta w_2$ Eqn 5f

<u>Inputs connected to hidden neuron 2</u>

$$\left(\frac{\partial C}{\partial b_2}\right) = \left(\frac{\partial C}{\partial a_2}\right)\left(\frac{\partial a_2}{\partial z_2}\right)\left(\frac{\partial z_2}{\partial b_2}\right) \text{ where } \left(\frac{\partial z_2}{\partial b_2}\right) = 1 \;\&\; \left(\frac{\partial a_2}{\partial z_2}\right) = \sigma'(z_2) = \frac{e^{z_2}}{(1+e^{z_2})^2}$$ Eqn 6a

$$\left(\frac{\partial C}{\partial w_3}\right) = \left(\frac{\partial C}{\partial a_2}\right)\left(\frac{\partial a_2}{\partial z_2}\right)\left(\frac{\partial z_2}{\partial w_3}\right) \text{ where } \left(\frac{\partial z_2}{\partial w_3}\right) = x_1$$ Eqn 6b

$$\left(\frac{\partial C}{\partial w_4}\right) = \left(\frac{\partial C}{\partial a_2}\right)\left(\frac{\partial a_2}{\partial z_2}\right)\left(\frac{\partial z_2}{\partial w_4}\right) \text{ where } \left(\frac{\partial z_2}{\partial w_4}\right) = x_2$$ Eqn 6c

Hence: $\Delta b_2 = -k_P\left(\sum_{i=1}^{N}\left(\frac{\partial C}{\partial b_2}\right)\right)/N \quad \& \quad b_{2(New)} = b_{2(Old)} + \Delta b_2$ Eqn 6d

$\Delta w_3 = -k_P\left(\sum_{i=1}^{N}\left(\frac{\partial C}{\partial w_3}\right)\right)/N \quad \& \; w_{3(New)} = w_{3(Old)} + \Delta w_3$ Eqn 6e

$\Delta w_4 = -k_P\left(\sum_{i=1}^{N}\left(\frac{\partial C}{\partial w_4}\right)\right)/N \quad \& \; w_{4(New)} = w_{4(Old)} + \Delta w_4$ Eqn 6f

Inputs connected to hidden neuron 3

$$\left(\frac{\partial C}{\partial b_3}\right) = \left(\frac{\partial C}{\partial a_3}\right)\left(\frac{\partial a_3}{\partial z_3}\right)\left(\frac{\partial z_3}{\partial b_3}\right) \text{ where } \left(\frac{\partial z_3}{\partial b_3}\right) = 1 \;\&\; \left(\frac{\partial a_3}{\partial z_3}\right) = \sigma'(z_3) = \frac{e^{z_3}}{(1+e^{z_3})^2}$$ Eqn 7a

$$\left(\frac{\partial C}{\partial w_5}\right) = \left(\frac{\partial C}{\partial a_3}\right)\left(\frac{\partial a_3}{\partial z_3}\right)\left(\frac{\partial z_3}{\partial w_5}\right) \text{ where } \left(\frac{\partial z_3}{\partial w_5}\right) = x_1$$ Eqn 7b

$$\left(\frac{\partial C}{\partial w_6}\right) = \left(\frac{\partial C}{\partial a_3}\right)\left(\frac{\partial a_3}{\partial z_3}\right)\left(\frac{\partial z_3}{\partial w_6}\right) \text{ where } \left(\frac{\partial z_3}{\partial w_6}\right) = x_2$$ Eqn 7c

Hence: $\Delta b_3 = -k_P \left(\sum_{i=1}^{N} \left(\frac{\partial C}{\partial b_3}\right)\right)/N$ & $b_{3(New)} = b_{3(old)} + \Delta b_3$ Eqn 7d

$\Delta w_5 = -k_P \left(\sum_{i=1}^{N} \left(\frac{\partial C}{\partial w_5}\right)\right)/N$ & $w_{5(New)} = w_{5(New)} + \Delta w_5$ Eqn 7e

$\Delta w_6 = -k_P \left(\sum_{i=1}^{N} \left(\frac{\partial C}{\partial w_6}\right)\right)/N$ & $w_{6(New)} = w_{6(New)} + \Delta w_6$ Eqn 7f

Inputs connected to hidden neuron 4

$\left(\frac{\partial C}{\partial b_4}\right) = \left(\frac{\partial C}{\partial a_4}\right)\left(\frac{\partial a_4}{\partial z_4}\right)\left(\frac{\partial z_4}{\partial b_4}\right)$ where $\left(\frac{\partial z_4}{\partial b_4}\right) = 1$ & $\left(\frac{\partial a_4}{\partial z_4}\right) = \sigma'(z_4) = \frac{e^{z_4}}{(1+e^{z_4})^2}$ Eqn 8a

$\left(\frac{\partial C}{\partial w_7}\right) = \left(\frac{\partial C}{\partial a_4}\right)\left(\frac{\partial a_4}{\partial z_4}\right)\left(\frac{\partial z_4}{\partial w_7}\right)$ where $\frac{\partial z_4}{\partial w_7} = x_1$ Eqn 8b

$\left(\frac{\partial C}{\partial w_8}\right) = \left(\frac{\partial C}{\partial a_4}\right)\left(\frac{\partial a_4}{\partial z_4}\right)\left(\frac{\partial z_4}{\partial w_8}\right)$ where $\left(\frac{\partial z_4}{\partial w_8}\right) = x_2$ Eqn 8c

Hence: $\Delta b_4 = -k_P \left(\sum_{i=1}^{N} \left(\frac{\partial C}{\partial b_4}\right)\right)/N$ & $b_{4(New)} = b_{4(Old)} + \Delta b_4$ Eqn 8d

$\Delta w_7 = -k_P \left(\sum_{i=1}^{N} \left(\frac{\partial C}{\partial w_7}\right)\right)/N$ & $w_{7(New)} = w_{7(Old)} + \Delta w_7$ Eqn 8e

$\Delta w_8 = -k_P \left(\sum_{i=1}^{N} \left(\frac{\partial C}{\partial w_8}\right)\right)/N$ & $w_{8(New)} = w_{8(Old)} + \Delta w_8$ Eqn 8d

In this way, Equations 2–8 are trained to minimize the cost function between the real and predicted output signals to almost zero and to obtain optimum values for arbitrary constants.
